# Effects of incretin treatment on cardiovascular outcomes in diabetic STEMI-patients with culprit obstructive and multivessel non obstructive-coronary-stenosis

**DOI:** 10.1186/s13098-017-0304-3

**Published:** 2018-01-03

**Authors:** Raffaele Marfella, Celestino Sardu, Maria Luisa Balestrieri, Mario Siniscalchi, Fabio Minicucci, Giuseppe Signoriello, Paolo Calabrò, Ciro Mauro, Gorizio Pieretti, Antonino Coppola, Gianfranco Nicoletti, Maria Rosaria Rizzo, Giuseppe Paolisso, Michelangela Barbieri

**Affiliations:** 1 Department of Medical, Surgical, Neurological, Aging and Metabolic Sciences, Università degli Studi della Campania “Luigi Vanvitelli”, Caserta, Italy; 2 Department of Biochemistry, Biophysics and General Pathology, Università degli Studi della Campania “Luigi Vanvitelli”, Caserta, Italy; 3 Department of Cardiology, Hospital Cardarelli, Naples, Italy; 4 Department of Mental Health and Public Medicine, Section of Statistic, Università degli Studi della Campania “Luigi Vanvitelli”, Caserta, Italy; 5 Department of Cardio-Thoracic and Respiratory Sciences, Università degli Studi della Campania “Luigi Vanvitelli”, Caserta, Italy; 6 Multidisciplinary Department of Surgical and Dental Specialties, Università degli Studi della Campania “Luigi Vanvitelli”, Caserta, Italy; 7 Cardiovascular Department, Hospital of Misericordia, Sorrento, Italy

**Keywords:** Type 2 diabetes, STEMI, Non-obstructive coronary stenosis

## Abstract

**Background:**

No proper data on prognosis and management of type-2 diabetic ST elevation myocardial infarction (STEMI) patients with culprit obstructive lesion and multivessel non obstructive coronary stenosis (Mv-NOCS) exist. We evaluated the 12-months prognosis of Mv-NOCS-diabetics with first STEMI vs.to non-diabetics, and then Mv-NOCS-diabetics previously treated with incretin based therapy vs. a matched cohort of STEMI-Mv-NOCS never treated with such therapy.

**Methods:**

1088 Patients with first STEMI and Mv-NOCS were scheduled for the study. Patients included in the study were categorized in type 2 diabetics (n 292) and non-diabetics (n 796). Finally, we categorized diabetics in current-incretin-users (n 76), and never-incretin-users (n 180). The primary end point was all cause deaths, cardiac deaths, and major adverse cardiac events (MACE) at 12 months of follow up.

**Results:**

The study results evidenced higher percentage of all cause deaths (2.2% vs. 1.1%, p value 0.05), cardiac deaths (1.6% vs. 0.5%, p value 0.045), and MACE (12.9% vs. n 5.9%), p value 0.001) in diabetic vs. no diabetic patients at 12 months follow up. Among diabetic patients, the current vs never-incretin-users, did not present a significant difference about all cause of deaths, and cardiac deaths through 12-months. The MACE rate at 1 year was 7.4% in diabetic incretin-users STEMI Mv-NOCS patients vs. 12.9% in diabetic never-incretin-users STEMI-Mv-NOCS patients (p value 0.04). In a risk-adjusted hazard analysis, MACE through 12 months were lower in diabetic STEMI-Mv NOCS incretin-users vs never-incretin-users patients (HR 0.513, CI [0.292–0.899], p 0.021). Consequently, lower levels of glucagon-like peptide 1(GLP-1) were predictive of MACE at follow up (HR 1.528, CI [1.059–2.204], p 0.024).

**Conclusion:**

In type 2 diabetic patients with STEMI-Mv-NOCS, we observed higher incidence of 1-year mortality and adverse cardiovascular outcomes, as compared to non-diabetic STEMI-Mv-NOCS patients. In diabetic patients, never-incretin-users have worse prognosis as compared to current-incretin-users.

*Trail registration* Clinical trial number: NCT03312179, name of registry: clinicaltrialgov, URL: clinicalltrialgov.com, date of registration: September 2017, date of enrollment first participant: September 2009

## Background

In general population, non-obstructive (< 50% stenosis diameter and flow fractional reserve > 0.80) non-infarcted related coronary diseases was common among patients presenting with ST-segment elevation myocardial infarction (STEMI), and were no associated with a significant increase in mortality [1]. In diabetic patients, there is a higher prevalence of multivessel disease, and of non obstructive coronary artery lesions [2, 3]. To date, STEMI diabetic patients with culprit obstructive lesion and multivessel non obstructive coronary stenosis (Mv-NOCS) represent a conundrum because no proper data regarding their prognosis and management exist. So far, incretin-based therapies have shown a broad range of unique cardiovascular actions translating into cardiovascular protection [4]. Therefore, given the paucity of data in this setting, we evaluated the 12-months prognosis of Mv-NOCS-diabetics with STEMI as compared with a matched cohort of non-diabetic patients. In this research we studied clinical outcomes after first STEMI event in STEMI-Mv-NOCS diabetics vs. non-diabetics, and then divided in diabetic incretin- users vs. diabetic never-incretin-users. As first, we compared number all cause of deaths, cardiac deaths, and of major adverse cardiac events (MACE) through 12 months in diabetic STEMI-Mv-NOCS patients vs. non-diabetic STEMI-Mv-NOCS patients. Secondary, we divided diabetic STEMI-Mv-NOCS incretin users vs. never-incretin-users, and we assessed all cause deaths, cardiac deaths, and MACE through 12-months of follow up. Our study hypothesis was that, diabetics STEMI-Mv-NOCS may have worse prognosis after first STEMI event as compared to non diabetics. Secondary, STEMI-Mv-NOCS diabetics current-incretin-users may present a significantly lower rate of MACE through 12 months as compared to a matched cohort of STEMI-Mv-NOCS-diabetics never treated with such therapy. Therefore, incretin therapy may represent a validate and innovative treatment to reduce worse prognosis in a population of STEMI-Mv-NOCS diabetics. Indeed, incretin therapy may improve clinical outcomes, ameliorating the prognosis of STEMI-Mv-NOCS diabetic patients.

## Methods

Consecutive 796 non diabetic and 292 diabetic patients with first STEMI and no-altered fractional flow reserve (FFR > 0.80) of Mv-NOCS (20–49% luminal stenosis), referred for coronary angiography within 12 h of clinical presentation of the clinical event, were entered in a database prospectively. STEMI was diagnosed according to international guidelines by evidence of myocardial injury (defined as an elevation of cardiac troponin values with at least one value above the 99th percentile upper reference limit), associated to symptoms consistent with myocardial ischemia, as persistent chest discomfort or other symptoms suggestive of ischemia (shortness of breath, nausea/vomiting, fatigue, palpitations, or syncope), and ST-segment elevation in at least two contiguous leads ≥ 2.5 mm in men < 40 years, ≥ 2 mm in men ≥ 40 years, or ≥ 1.5 mm in women in leads V2–V3 and/or ≥ 1 mm in the other leads [5]. In these patients, we performed an early, and immediate coronary angiography followed by percutaneous coronary intervention to have a rapid restoration of epicardial blood flow in the infarct related artery [5].Therefore, patients with no coronary disease detected by coronary angiography, presence of obstructive and Mv-obstructive stenosis, left ventricular ejection fraction less than 25%, previous myocardial infarction, previous PCI or/and coronary by-pass grafting, Tako-tsubo cardiomyopathy, myocarditis, acute or chronic infection or inflammatory diseases, hematologic disorder, malignancies, end-stage liver or renal disease, and use of steroid therapy or chemotherapy were excluded. Subjects were categorized in non-diabetic and diabetic patients [6]. Furthermore, the diabetic patients answered a specific questionnaire about medicines used for diabetes treatment before the beginning of the study, the dates of the beginning and the end of treatment, the route of administration, and the duration of use. Information from the medicine inventory during the study and this specific questionnaire was used to classify the subjects. The patients with diabetes who never used incretin, such as glucagon-like peptide 1 (GLP-1) agonists and dipeptidyl peptidase-4 (DPP-4) inhibitors, were classified as “never-incretin-users.” The patients with diabetes who had already used, for at least 6 months, GLP-1 agonists or DPP-4 inhibitors were classified as “current incretin-users”. Therefore, upon emergency wards admission, all patients were assigned to undergo prompt coronary angiography. This was a multi center prospective “real world” study conducted at University of Campania “Luigi Vanvitelli”, Cardarelli hospital, and Monaldi hospital (Naples, Italy), between July 2009 and July 2016. The study was conducted in accordance with the Declaration of Helsinki. The Ethics Committees of all participating institutions approved the protocol (Ethic Committee University of Campania “Luigi Vanvitelli” number: 1177). All patients were informed about the study nature, and gave their written informed, and signed consent to participate in the study. The study was retrospectively registered.

### Study protocol

#### Laboratory analysis

After an overnight fast, plasma glucose and HbA1c levels were measured by enzymatic assays in the hospital chemistry laboratory. GLP-1 levels (Active GLP-1 [7-36] Specific ELISA Kit; Epitope Diagnostics) were measured after an overnight fast (at 8:00 A.M.) and after breakfast in diabetic patients. A standardized hospital breakfast for ACS patients contained 419 kcal (57% carbohydrate, 17% protein, and 26% fat). After breakfast, blood samples for the measurement of GLP-1 were obtained every 30 min over a 2-h period. The mean of the four GLP-1 evaluations was defined as the postprandial GLP-1 value. The standardized meal tolerance test and baseline evaluations were performed 5 days after STEMI.

#### Inflammatory markers

Routine analyses and inflammatory status, as ratio between macrophages 1 (CD68) and macrophages 2 (soluble-CD163) (M1/M2 ratio), and high sensitivity C-reactive protein (hs-CRP), were obtained on admission before coronary angiography and before full medical therapy was started.

#### Quantitative coronary angiography

Upon emergency wards admission, all patients were assigned to undergo prompt coronary angiography. The analyses of all angiographic data before were performed by three interventional cardiologists (M.F., M.C. and C.P.), and followed by percutaneous coronary intervention (PCI) with angioplasty and direct stenting of culprit vessel lesion [6]. Coronary stenting of culprit coronary vessel lesion was the technique of choice for all admitted patients [6]. Therefore, admitted STEMI diabetic and non diabetic patients received preferably primary PCI (92%, n 1001). On other hand, a low percentage of STEMI patients (8%, n 87) were diagnosed in non-PCI-capable hospitals, and they did not receive primary PCI. In these patients, physicians performed a thrombolytic reperfusion therapy. Moreover, 69 patients (79%) received rescue PCI, and 56 patients (65%) were treated by stent implantation. After that, these cardiologists blinded to patient categorization, reviewed selecting cases with Mv-NOCS, as coronary vessels with no-altered fractional flow reserve (FFR > 0.80), and associated to 20–49% luminal stenosis [5, 7].

### Coronary care unit/intensive cardiac care unit

All treated patients were then monitored and managed in Intensive Care Unit following reperfusion, by continuous monitoring, and specialized care [6] for STEMI and related acute complications (arrhythmias, heart failure, etc.) treatment.

#### Echocardiographic assessment

At admission patients underwent two-dimensional echocardiography as previously described [8]. This exam was used to asses heart chambers morphology, volumetry, wall contraction, cardiac valves morphology and function, and ejection fraction [8]. To asses heart chambers wall contractility we used scheme as previously described [8]. This exam was used at admission to confirm STEMI diagnosis, and during follow up to stage STEMI disease progression (6 and 12 months after STEMI).

#### Follow-up

After discharge from the hospital, all patients were managed and followed quarterly for 12 months after event, as outpatients, to perform clinical evaluation, routine analyses and cardiovascular evaluation (ECG, exercise ECG, echocardiography, exercise myocardial scintigraphy), as well as with the goal to maintain HbA1c level at < 7%, fasting blood glucose level of 90–140 mg/dl, and post-prandial blood glucose level of < 180 mg/dl. The mean follow-up was 16 ± 3 months. Follow-up visits were performed in our outpatients clinic.

#### Cardiovascular endpoints

The study end point was all cause deaths, cardiac deaths, and major adverse cardiac events (MACE) at 12 months follow up.

### Statistical analysis

SPSS version 23.0 (IBM statistics) was used for all statistical analyses. Categorical variables were presented as frequencies (percentages) and continuous variables as mean ± SD. For the general population of diabetics and non diabetics we calculated a sample size using a power of 80% and confidence of 95%. For comparison among diabetic never-incretin-users and diabetic current-incretin-users, a propensity score matching (PSM) was developed from the predicted probabilities of mortality and MACE by a multivariable logistic regression model. Diabetic never-incretin-users were matched to diabetic current-incretin-users on the basis of PSM. In all matched patients, the balancing property was satisfied. Overall survival and event-free survival were presented using Kaplan–Meier survival curves and compared using the log-rank test. Univariable Cox models were then used to compare event risks. Within all the diabetic and non-diabetic groups, all cause of deaths, cardiac deaths, and MACE were assessed by using multivariable Cox models with adjustment for statistically different variables at baseline and follow-up: hypertension, dyslipidemia, current smoking, ace-inhibitors, calcium inhibitors, thiazide diuretics, aspirin, statin, BMI, heart rate, HDL-cholesterol, LDL-cholesterol, triglycerides levels, hs-CRP, M1/M2, and GLP-1 levels. The resulting hazard ratios (HRs) and 95% confidence intervals (CIs) were reported. To investigate the effects of GLP1 levels on cardiovascular endpoints, we evaluated STEMI outcomes at 1-year follow-up stratified by GLP-1 quartiles. A 2-tailed p value < 0.05 was considered statistically significant.

## Results

**Fig. 1:**
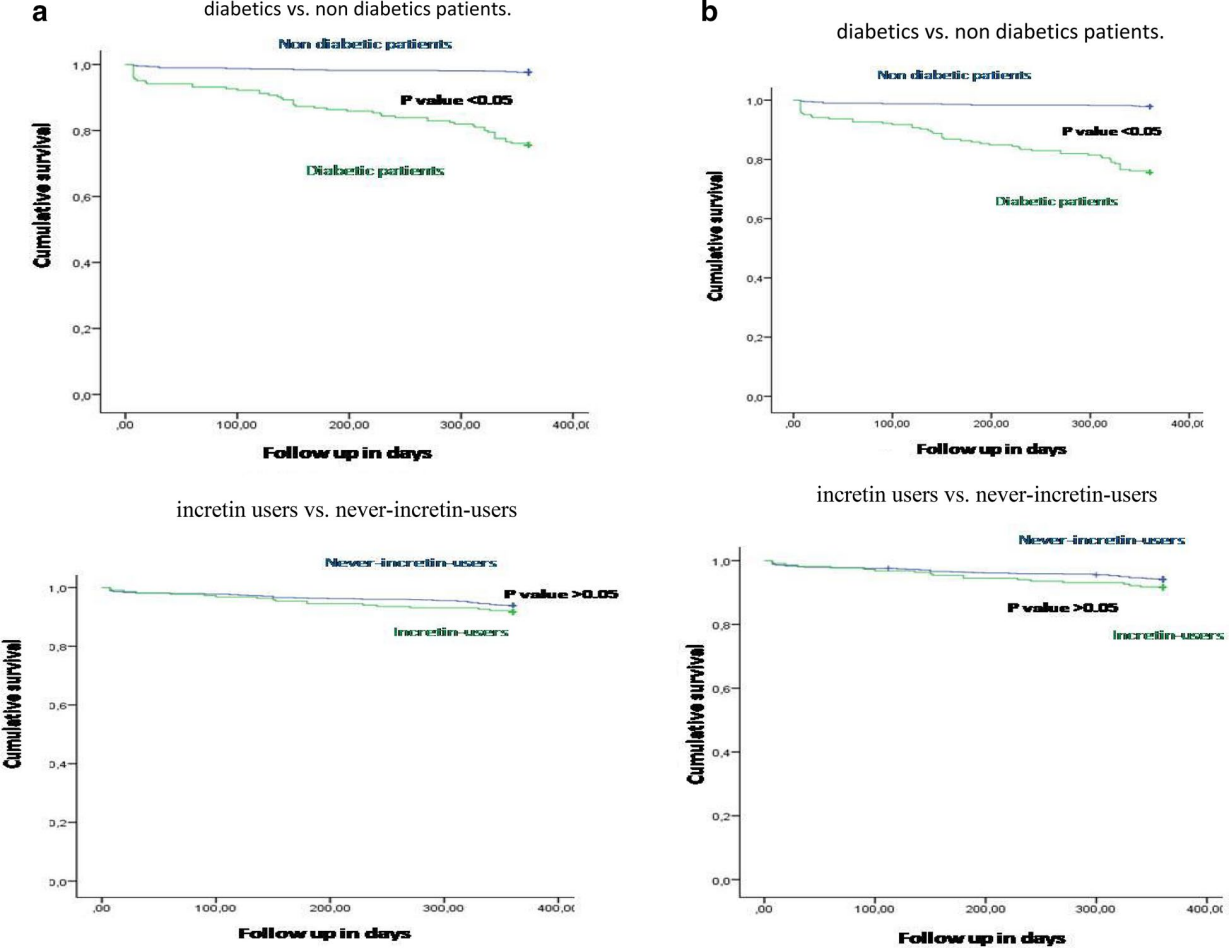
**a** Kaplan–Meier curve for all cause deaths. In left part all cause deaths cumulative survival curve at 360 days follow up comparing diabetic (green color) vs. non diabetic patients (blue color). In right part all cause deaths cumulative survival curve at 360 days follow up comparing diabetic incretin users (green color) vs. diabetic never-incretin-users patients (blue color). There is a statistical significant higher number of events comparing diabetic vs.non diabetic patients (p value < 0.05). **b** Kaplan–Meier curve for cardiac deaths. Kaplan–Meier curve for cardiac deaths. In left part all cause deaths cumulative survival curve at 360 days follow up comparing diabetic (green color) vs. non diabetic patients (blue color). In right part all cause deaths cumulative survival curve at 360 days follow up comparing diabetic incretin users (green color) vs. diabetic never-incretin-users patients (blue color). There is a statistical significant higher number of events comparing diabetic vs.non diabetic patients (p value < 0.05)

**Fig. 2:**
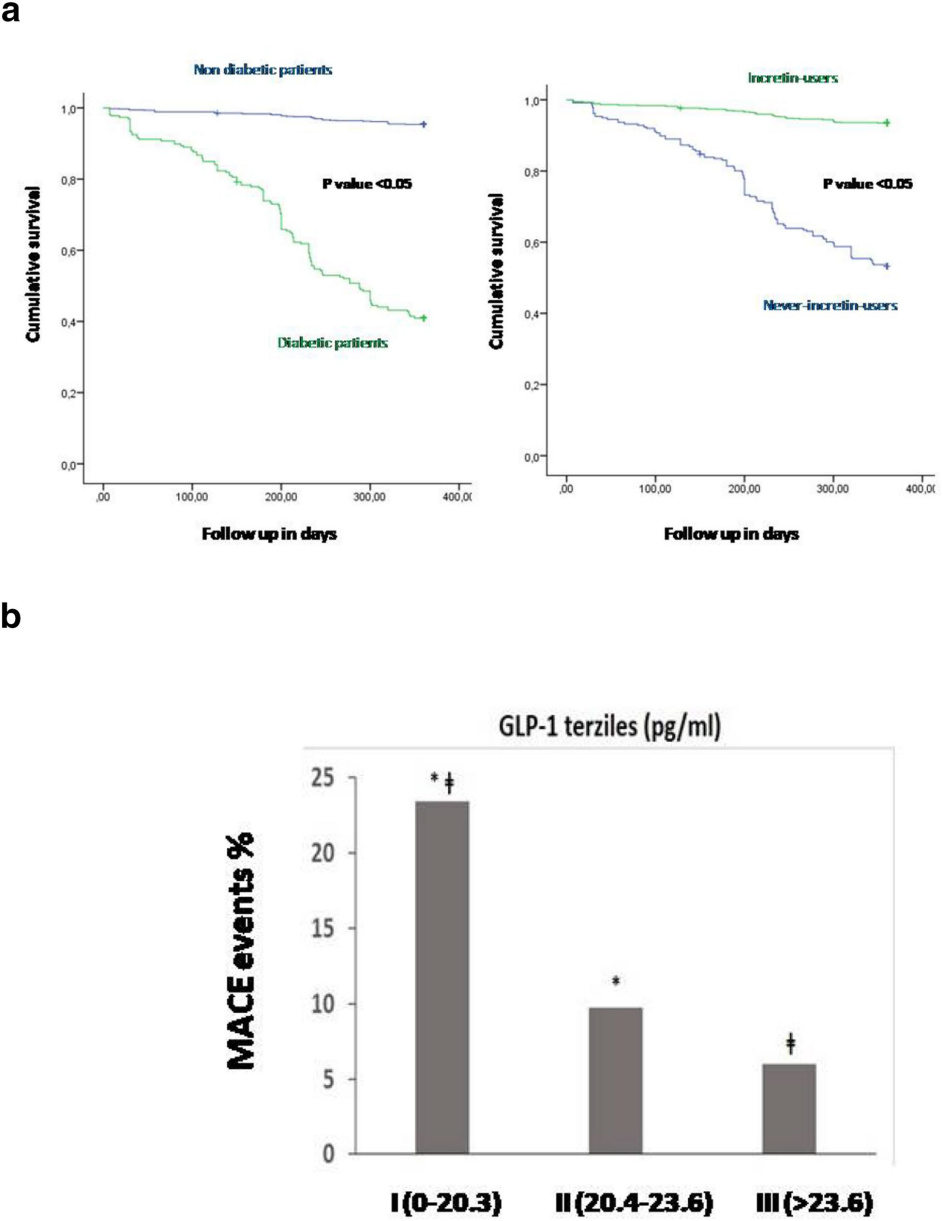
**a** Kaplan–Meier curve for major adverse cardiac events (MACE). Kaplan–Meier curve for major adverse cardiac events (MACE). In left part MACE cumulative survival curve at 360 days follow up comparing diabetic (green color) vs. non diabetic patients (blue color). In right part MACE cumulative survival curve at 360 days follow up comparing diabetic incretin users (green color) vs. diabetic never-incretin-users patients (blue color). There is a statistical significant higher number of events comparing diabetic vs.non diabetic patients, and incretin-users vs. never-incretin-users (p value < 0.05). **b** Major adverse cardiac events (MACE) outcome at 1-year follow-up stratified by GLP-1 terziles. After breakfast, blood samples for the measurement of GLP-1 were obtained every 30 min over a 2-h period. The mean of the four GLP-1 evaluations was defined as the postprandial GLP-1 value. Diabetic patients are divided by GLP-1 values in three subgroup: I terzile with GLP-1 values < 20.3 ng/ml; II terzile with GLP-1 values between 20.4 and 23.6 ng/ml; III terzile with GLP-1 values > 23.6 ng/ml. Major number of MACE are associated to I GLP-1 terzile. I GLP-1 terzile is statistical significant higher vs. II and III GLP-1 terzile (respectively marked with symbol *, and ǂ)

Between July 2009 and July 2016, 769 non-diabetics and 292 diabetics (122 current-incretin-users: 26 treated with glucagon-like peptide-1 receptor agonist and 96 with dipeptidyl peptidase-4 inhibitor; 170 never-incretin-users) meet inclusion criteria among all patients admitted to emergency wards (Table 1). After PSM for metabolic and cardiovascular risk factors, 67 never-incretin-users and 67 current-incretin-users were matched. The matched cohorts had similar characteristics (Table 1). The mean (± SD) duration of incretin treatment was 27 ± 2.2 months. Basal and post prandial GLP-1 levels were higher in current incretin users compared with never incretin users (p < 0.01) (Table 1). M1/M2 ratio and hs-CRP levels were higher in diabetic patients compared to non-diabetic patients (p < 0.01) (Table 1). The all cause deaths at 1 year was 2.2% in all diabetic STEMI-Mv-NOCS patients vs. 1.1% in non-diabetic STEMI-Mv-NOCS patients (p 0.05) (Table 2, Fig. 1a). Cardiac deaths at 1 year was 1.6% in all diabetic STEMI-Mv-NOCS patients vs. 0.5% in non-diabetic STEMI-Mv-NOCS patients (p 0.045) (Table 2, Fig. 1b). The MACE rate at 1 year was 12.9% in all diabetic STEMI-Mv-NOCS patients vs. 5.9% in non-diabetic STEMI-Mv-NOCS patients (p < 0.01) (Table 2, Fig. 2a). Among diabetic patients, the current vs never-incretin-users, did not present a significant difference about all cause of deaths, and cardiac deaths through 12-months (Table 2, Fig. 1a, b). The in-hospital MACE rate did not differ between non-diabetic patients (1.92%) and all diabetic patients (2.26%). The MACE rate at 1 year was 7.4% in diabetic incretin-users STEMI-Mv-NOCS patients vs. 12.9% in diabetic never-incretin-users STEMI-Mv-NOCS patients (p 0.04) (Table 2, Fig. 2a). In a risk-adjusted hazard analysis, STEMI-Mv-NOCS diabetic patients vs. STEMI-Mv-NOCS non diabetic patients exhibited a higher risk of all cause deaths (HR 2.172, 95% CI [1.225–3.925], p value 0.010), cardiac deaths (HR 2.253, 95% CI [1.245–4.078], p value 0.007), and MACE (HR 1.962, 95% CI [1.124–3.422], p value 0.018) (Table 3). Incretin therapy did not have effect on all cause deaths, and cardiac mortality. On the contrary, incretin therapy reduced the risk to have MACE at follow up (HR 0.565, CI 95% [0.387–0.824], p value 0.003) (Table 3, Fig. 2a). Finally, to translate the effects of incretin therapy on GLP-1 levels in real clinical endpoints, we evaluated STEMI outcomes at 1-year follow-up stratified GLP-1 quartiles in all study population. As evidenced in Fig. 2b, patients with higher GLP-1 levels (I terzile of GLP-1 values < 20 pg/ml) had lower number of events. Parallel to this study result, lower baseline value of GLP-1 (GLP-1 I terzile, values < 20 pg/ml), resulted in increased risk of MACE at 12 months follow up (HR 1.528, 95% CI [1.059–2.204], p value 0.024) (Table 3).

**Table 1:** Baseline clinical characteristics, angiographic and procedural data of patients with STEMI and multivessel non-obstructive coronary stenosis (NOCS) respecting the inclusion criteria

	Non-diabetic patients	Diabetic patients	p	PSM-diabetic never incretin users	PSM-diabetic current incretin users	p
N	796	292		67	67	
Mean age (years)	65.5 ± 5.9	64.9 ± 9.5	0.184	64.4 ± 5.7	65.3 ± 5.7	0.289
Sex (M/F)	446/350	157/135	–	37/30	39/28	–
BMI (kg/m^2^)	27.5 ± 1.1	29.0 ± 1.9	0.001	29.3 ± 2.1	29.2 ± 2.8	0.732
Diabetes duration (years)	–	16.6 ± 3.4	–	16.2 ± 3.1	16.8 ± 3.4	0.299
Systolic blood pressure (mmHg)	126.9 ± 9.2	125.9 ± 10.4	0.129	124.5 ± 10.4	125.8 ± 11.3	0.507
Diastolic blood pressure (mmHg)	79.7 ± 8.6	79.1 ± 6.7	0.294	79.8 ± 6.6	79.8 ± 6.6	0.727
Heart rate (bpm)	85.1 ± 7.4	85.9 ± 9.1	0.148	86.3 ± 11.2	86.7 ± 8.5	0.789
Grace score, n (%)						
I	532 (66.8)	204 (69.9)	0.192	42 (62.3)	38 (56.8)	0.192
II	209 (26.3)	76 (26.0)	0.503	21 (31.3)	24 (35.8)	0.357
III	45 (5.7)	14 (4.8)	0.350	4 (5.9)	5 (7.5)	0.341
Risk factors						
Stress hyperglycemia, n (%)	39 (4.9)	91 (31.2)	0.001	30 (44.8)	27 (40.3)	0.363
Hypertension, n (%)	427 (53.6)	230 (78.8)	0.001	55 (82.1)	50 (74.6)	0.201
Hyperlipemia, n (%)	214 (26.9)	103 (35.3)	0.005	38 (56.7)	39 (58.2)	0.500
Cigarette smoking, n (%)	101 (12.7)	25 (8.6)	0.035	11 (16.4)	10 (14.9)	0.500
Active treatments						
β-blokers, n (%)	266 (33.4)	106 (36.3)	0.207	39 (58.2)	33 (49.3)	0.193
ACE inhibitors, n (%)	224 (28.1)	61 (20.9)	0.009	20(29.9)	17 (25.4)	0.139
Angiotens inreceptorblokers, n (%)	289 (36.3)	127 (43.5)	0.019	29(43.3)	29 (43.3)	0.569
Calcium inhibitor, n (%)	197 (24.7)	51 (17.5)	0.006	8 (11.9)	12 (17.9)	0.234
Nitrate, n (%)	396 (49.7)	141 (48.3)	0.360	40 (59.7)	35 (52.2)	0.243
Statins, n (%)	179 (22.5)	83 (28.4)	0.027	32 (47.8)	29 (43.3)	0.364
Thiazidediuretic, n (%)	88 (11.1)	16 (5.5)	0.003	7 (10.4)	8 (11.9)	0.500
Insulin, n (%)	–	67 (23.5)	–	27 (25.2)	26 (24.3)	0.507
Meftformin, n (%)	–	250 (87.7)	–	94 (87.8)	95 (88.8)	0.124
Sulfonylureas, n (%)	–	57 (20.0)	–	22 (20.1)	23 (21.5)	0.177
Acarbose, n (%)	–	31 (10.9)	–	12 (11.2)	11 (10.3)	0.252
Thiazolidinediones, n (%)	–	17 (5.9)	–	6 (5.6)	7 (6.5)	0.098
GLP-1agonists, n (%)	–	51 (17.9)	–	–	23 (21.5)	–
DPP-4inhibitors, n (%)	–	142 (49.8)	–	–	84 (78.5)	–
Aspirin, n (%)	228 (28.6)	111 (38.0)	0.002	30 (44.8)	27 (40.3)	0.363
Thienopyridine, n (%)	81 (10.2)	31 (10.6)	0.455	8 (11.9)	6 (9.0)	0.389
Low-molecular heparin, n (%)	38 (4.8)	15 (5.1)	0.456	4 (6.0)	2 (3.0)	0.340
Vitamin-Kantagonist, n (%)	28 (3.5)	6 (2.1)	0.150	2 (3.0)	2 (3.0)	0.248
Laboratory analyses						
Plasma glucose (mg/dl)	109.6 ± 17.5	201.8 ± 25.7	0.001 199.3 ± 29.6 202.9 ± 24.1 0.426	199.3 ± 29.6	202.9 ± 24.1	0.426
HbA1c (%)	5.6 ± 1.1	8.7 ± 0.8	0.001	8.8 ± 0.72	8.9 ± 0.85	0.237
Cholesterol (mg/dl)	205.5 ± 19.4	206.8 ± 24.6	0.361	204.0 ± 25.0	207.3 ± 19.0	0.386
LDL-cholesterol (mg/dl)	130.4 ± 19.5	133.3 ± 23.9	0.039	130.0 ± 24.2	134.0 ± 24.1	0.284
HDL-cholesterol (mg/dl)	38.6 ± 3.2	36.9 ± 3.5	0.001	37.2 ± 3.8	36.8 ± 3.5	0.556
Triglycerides (mg/dl)	182.5 ± 19.5	188.8 ± 24.4	0.001	189.6 ± 23.5	188.2 ± 23.6	0.735
Creatinine (mg/dl)	0.99 ± 0.15	0.98 ± 0.15	0.471	0.95 ± 0.17	0.98 ± 0.16	0.204
hs-cTnT (ng/l)	14.8 ± 1.75	14.9 ± 2.5	0.216	15.0 ± 2.6	14.7 ± 1.5	0.557
HsC-reactive protein	1.9 ± 0.3	4.0 ± 1.9	0.001	4.1 ± 0.9	1.2 ± 0.7	0.001
M1/M2ratio	4.9 ± 2,6	15.8 ± 6.2	0.001	12.8 ± 2.4	5.5 ± 1.9	0.001
BasalGLP-1 (pmol/L)	7.1 ± 1.1	4.9 ± 1.6	0.033	11.0 ± 2.1	15.3 ± 4.8	0.001
PostprandialGLP-1 (pmol/L)	26.8 ± 4.1	13.9 ± 6.7	0.001	11.3 ± 2.2	15.3 ± 4.8	0.001
LVEF, n (%)						
> 50%	516 (64.8)	198 (67.8)	0.199 38 (56.7) 42 (62.7) 0.299	38 (56.7)	42 (62.7)	0.299
41–50%	233 (29.3)	81 (27.7)	0.339	25 (37.3)	20 (29.9)	0.232
25–40%	47 (5.9)	13 (4.5)	0.220	4 (6.0)	5 (7.5)	0.500
Procedural data						
Symptom onset to angiography, h	7.1 ± 1.2	6.9 ± 0.8	0.004	7.0 ± 0.8	6.9 ± 0.8	0.180
Insulin infusion time, min	–	41.6 ± 3.1	– 41.6 ± 3.1/42.6 ± 2.9 43.2 ± 3.2 0.316	42.6 ± 2.9	43.2 ± 3.2	0.316
Angiographic data						
Quantitative angiographic data						
*Culprit obstructive lesion*						
Lesion length, mm	20.2 ± 2.12	20.9 ± 2.02	0.376 20.6 ± 1.6 20.2 ± 2.4 0.522	20.6 ± 1.6	20.2 ± 2.4	0.522
Reference diameter, mm	2.7 ± 0.3	2.8 ± 0.4	0.087	2.8 ± 0.5	2.7 ± 0. 6	0.485
MLD,	1.0 ± 0.21	1.1 ± 0.12	0.121	1.1 ± 0.13	1.1 ± 0.11	0.807
MLD post (in-stent). mm	2.7 ± 1.6	2.7 ± 0.3	0.335			
*No*-*culprit NOCS*						
*Number of vessels, n (%)*						
1-VD	346 (43.5)	135 (46.2)	0.228	66 (61.7)	68 (63.5)	0.218
2-VD	257 (32.3)	80 (27.4)	0.070	36 (33.6)	35 (32.7)	0.355
3-VD	193 (24.2)	77 (26.4)	0.260	5 (4.7)	4 (3.7)	0.335
Stenosis (%)	43.8 ± 2.1	44.13.2	0.092	43.9 ± 1.9	44.5 ± 2.2	0.124
Lesion length, mm	15.8 ± 3.1	15.8 ± 3.9	0.863	15.6 ± 3.5	16.1 ± 3.8	0.387
Reference diameter, mm	2.8 ± 0.4	2.8 ± 0.5	0.283	2.7 ± 0.51	2.9 ± 0.56	0.104
MLD, mm	1.8 ± 1.5	1.83 ± 1.14	0.733	1.8 ± 1.35	1.9 ± 2.35	0.667
FFR, pd/pa	0.84 ± 0.028	0.82 ± 0.019	0.189	0.84 ± 0.017	0.83 ± 0.019	0.889

**Table 2:** Study endpoints in diabetics vs. overall study population, and in incretin-users vs. never-incretin-users

	Non-diabetic patients	Diabetic patients	p	PSM-diabetic never incretinusers	PSM-diabetic current incretinusers	p
N	796	292		67	67	
All cause deaths	9 (1.1%)	6 (2.2%)	0.05	3 (4.5%)	3 (4.5%)	/
Cardiac deaths	39 (0.5%)	5 (1.6%)	0.045	3 (4.5%)	2 (3.0%)	/
MACE	47 (5.9%)	38 (12.9%)	0.001	9 (12.9%)	5 (7.4%)	0.04

MACE is for major adverse cardiac events; the symbol “/” is indicating not statistical significant (p value > 0.05)

**Table 3:** Univariate and multivariate analysis of factors to predict all cause deaths at follow up

	Univariate analysis		Multivariate analysis	
	HR (95% CI)	p value	HR (95% CI)	p value
A. Multivariate cox regression analysis for parameters associated with all cause deaths				
Diabetes	1.603 [0.919–2.797]	0.097	2.172 [1.225–3.925]	0.010*
Hypertension	0.658 [0.407–1.064]	0.088	1.519 [0.926–2.492]	0.098
Dyslipidemia	0.906 [0.531–1.544]	0.716	1.232 [0.715–2.122]	0.452
Smoke	1.784 [1.119–2.844]	0.045	1.133 [0.694–1.851]	0.621
LVEF < 50%	1.176 [0.727–1.901]	0.51	0.901 [0.558–1.453]	0.669
Bas.Lesion length	0.953 [0.849–1.070]	0.415	0.973 [0.866–1.092]	0.638
Obesity	0.964 [0.529–1.758]	0.906	0.952 [0.493–1.841]	0.885
Incretin	0.901 [0.528–1.535]	0.700	1.274 [0.664–2.447]	0.466
VD-3	0.859 [0.493–1.497]	0.592	1.284 [0.727–2.268]	0.389
HsCRP	1.188 [0.545–2.191]	0.665	0.623 [0.011–3.247]	0.858
M1/M2 ratio	0.958 [0.438–2.088]	0.913	1.136 [0.319–3.221]	0.922
LowGLP-1	0.901 [0.413–1.965]	0.793	3.714 [0.215–4.902]	0.922
B. Multivariate cox regression analysis is for parameters associated with cardiac deaths				
Diabetes	1.663 [0.951–2.908]	0.075	2.253 [1.245–4.078]	0.007*
Hypertension	0.705 [0.433–1.146]	0.159	1.438 [0.871–2.375]	0.155
Dyslipidemia	0.959 [0.561–1.642]	0.88	1.174 [0.679–2.031]	0.565
Smoke	1.757 [1.092–2.828]	0.02	1.024 [0.626–1.675]	0.924
LVEF < 50%	1.049 [0.653–1.386]	0.842	0.877 [0.538–1.430]	0.601
Bas.Lesion length	0.942 [0.837–1.061]	0.326	0.961 [0.853–1.081]	0.638
Obesity	1.017 [0.556–1.860]	0.956	0.859 [0.441–1.673]	0.656
Incretin	0.816 [0.466–1.427]	0.475	1.531 [0.762–3.074]	0.231
VD-3	0.838 [0.473–1.484]	0.544	1.285 [0.716–2.308]	0.401
HsCRP	1.244 [0.569–2.117]	0.584	7.546 [0.716–8.403]	0.859
M1/M2 ratio	1.003 [0.459–2.191]	0.994	1.107 [0.011–4.934]	0.991
LowGLP-1	0.944 [0.432–2.62]	0.885	4.029 [0.028–4.802]	0.823
C. Multivariate cox regression analysis is for parameters associated with major adverse cardiac events (MACE)				
Diabetes	0.952 [0.621–1.461]	0.822	1.962 [1.124–3.422]	0.018*
Hypertension	0.929 [0.678–1.274]	0.649	1.058 [0.751–1.490]	0.748
Dyslipidemia	0.899 [0.634–1.275]	0.55	1.148 [0.775–1.698]	0.748
Smoke	1.158 [0.839–1.598]	0.372	0.829 [0.578–1.190]	0.309
LVEF < 50%	0.994 [0.725–1.363]	0.969	0.277 [0.590–1.163]	0.829
Bas.Lesion length	1.032 [0.955-1.115]	0.048	1.023 [0.930–1.126]	0.637
Obesity	0.320 [0.235-0.437]	0.001	1.528 [0.509-2.204]	0.064
Incretin	0.257 [0.187–0.355]	0.001	0.565 [0.387–0.824]	0.003*
VD-3	1.806 [1.167–2.794]	0.008	1.173 [0.728–1.888]	0.513
HsCRP	35.947 [26.067–49.553]	0.001	1.938 [0.908–4.137]	0.087
M1/M2 ratio	0.019 [0.014–0.027]	0.001	0.773 [0.293–2.037]	0.603
LowGLP-1	0.018 [0.012–0.025]	0.001	1.528 [1.059–2.204]	0.024*

(A) Univariate and multivariate analysis of factors to predict all cause deaths at follow up. We have considered as statistical significant a p value < 0.005, with hazard ratio (HR) at 95% of confidence of interval (CI). At multivariable analysis the parameter associated with a statistical significant value (p value < 0.005) has been marked with the symbol*. Bas.Lesion length is indicating basal lesion length; HsCRP is for high sensitivity C reactive protein; Low GLP-1 is indicating lower terzile of GLP-1 (glucagon-like peptide 1) values, as < 20 pg/ml; LVEF is for left ventricle ejection fraction; M1/M2 ratio is the ration between macrophage 1 and macrophage 2 cells. VD-3 is indicating a multivessel coronary disease with 3 coronary vessels

(B) Univariate and multivariate analysis of factors to predict cardiac deaths at follow up. We have considered as statistical significant a p value < 0.005, with hazard ratio (HR) at 95% of confidence of interval (CI). At multivariable analysis is the parameter associated with a statistical significant p value (p value < 0.005) has been marked with the symbol*. Bas.Lesion length is indicating basal lesion length; HsCRP is for high sensitivity C reactive protein; Low GLP-1 (glucagon-like peptide 1) is indicating lower terzile of GLP-1values, as < 20 pg/ml; LVEF is for left ventricle ejection fraction; M1/M2 ratio is the ratio between macrophage 1 and macrophage 2 cells. VD-3 is indicating a multi vessel coronary disease with 3 coronary vessels

(C) Univariate and multivariate analysis of factors to major adverse cardiac events (MACE) at follow up. We have considered as statistical significant a p value < 0.005, with hazard ratio (HR) at 95% of confidence of interval (CI). At multivariable analysis is the parameter associated with a statistical significant value (p value < 0.005) has been marked with the symbol*. Bas.Lesion length is indicating basal lesion length; HsCRP is for high sensitivity C reactive protein; Low GLP-1 (glucagon-like peptide 1) is indicating lower terzile of GLP-1 values, as < 20 pg/ml; LVEF is for left ventricle ejection fraction; M1/M2 ratio is the ratio between macrophage 1 and macrophage 2 cells. VD-3 is indicating a multivessel coronary disease with 3 coronary vessels

## Discussion

The main results were as follows: first, in a contemporary sample of type 2 diabetic patients with STEMI-Mv-NOCS, we observed higher cumulative incidence of 1-year mortality and adverse cardiovascular outcomes as compared to non-diabetic STEMI-Mv-NOCS patients; second, in PSM diabetic patients, diabetic never-incretin-users have higher number of MACE as compared to diabetic current-incretin-users. The prognosis of patients with NOCS has been evaluated, by a recent study [9], which evidenced that among individuals without known CAD and obstructive CAD, non obstructive plaque presence enhances risk prediction of incident mortality. Moreover, [9, 10] among patients with type 2 diabetes, non obstructive and obstructive stable CAD were associated with higher rates of all-cause mortality and major adverse cardiovascular events at 5 years, and this risk was significantly higher than in non-diabetic subjects. However, these studies did not provide any evidence about the influence of STEMI-Mv-NOCS management on outcomes following the cardiac event in diabetic patients. In our study after STEMI, we observed an increased incidence of cardiovascular disease in STEMI-Mv-NOCS patients, both after adjustment for baseline, and follow up cardiovascular risk factors. In this context, the poor outcomes of diabetic STEMI-Mv-NOCS as compared to non-diabetic Mv-NOCS-STEMI, observed in our study, might be explained by an abruptly increment of atherosclerosis in diabetics as compared to a more slow progression of coronary atherosclerosis extension in non-diabetics [11]. In this scenario, the diabetic status may affects several pathogenetic mechanisms, that favor the plaque instability and subsequently plaque rupture in the absence of obstructive coronary stenosis, including inflammation, endothelial dysfunction with the inability to augment coronary flow in response to stress, and coronary vasospasm. Accordingly, our data evidenced more inflammatory cells and CPR levels in diabetic than in no-diabetic patients (Table 1). The present findings also show a protective effect of incretin therapies on cardiovascular outcomes in Mv-NOCS diabetic patients after STEMI. Without conditioning cardiac mortality, and all cause of deaths, incretin therapy may affect MACE at 12 months follow up. Indeed, diabetic patients treated with incretin therapies had the lowest incidence of cardiovascular events at the same level of blood glucose levels vs. never-incretin-users. In human randomized, double-blind clinical studies, DPP-4 inhibitors did not appear to reduce the risk of major adverse cardiovascular events among patients with type 2 diabetes without and with established cardiovascular disease [12–14]. However, definitive proof of an effect of DPP-4 inhibitors in patients with acute coronary syndrome, as well as in patients with DPP-4 inhibitors therapy before the cardiovascular event is currently lacking. In our study after STEMI, the 1-year follow-up results show a higher reduction in the MACE endpoint in patients previous treated with incretin as compared to patients without incretin-therapy despite a similar severity of atherosclerotic disease (coronary stenosis < 50%; FFR > 0.80) at baseline. Moreover, both at baseline and at follow-up the current-incretin-users presented lower levels of inflammatory cells, as reported by a M1/M2 ratio and inflammatory markers as CRP, and higher GLP-1 values (Table 1). Accordingly, human studies showed that sitagliptin, vildagliptin and exenatide [15–17], even at a single dose, exert a potent anti inflammatory effect, and that many of these effects were persistent over a period of 12 weeks, thus suggesting that the anti-inflammatory effects of GLP-1–based therapies could help to reduce atherosclerosis progression. This concept has been recently investigated by authors [18], reporting that, in acute coronary syndromes, the cardiovascular outcomes were strictly correlated to postprandial GLP-1 levels independently from endogenous (DPP-4 inhibitors) vs exogenous (GLP-1 agonist) treatments. Therefore, patients assigned to incretin therapy may have a lesser plaque progression to an unstable phenotype, than patients assigned to other anti-diabetic therapies [18]. In our study we evidenced that patients with higher GLP-1 levels had lower number of events. Moreover, we may report a protective cardiovascular effect of GLP-1 agonist therapy on atherosclerotic plaques of patients with diabetes, as previously described [19]. However, these results may be due to the small sample size of study population, and the short time of follow-up duration, and future clinical trial have to assess this research topic.

## Conclusion

The novelty of this research is to show “real world data” about clinical outcomes in diabetic STEMI-patients with culprit obstructive lesion and Mv-NOCS treated by incretins vs. standard hypoglycemic drugs. Moreover, diabetics current-incretin-users vs. never-incretin-users presented a significantly lower rate of MACE through 12 months, as represented by the evident significant abrupt decreasing of Kaplan–Meier survival curves free from MACE (Fig. 1). This study result supports incretin therapy as the best treatment of diabetics STEMI-Mv-NOCS patients. Therefore, incretin effect on the control of hyperglycemia homeostasis may be associated to other pleiotropic effects, than playing a decisive rule in the control of atherosclerotic plaque progression, and functionality in diabetic STEMI-Mv-NOCS patients. In conclusion, diabetic STEMI-Mv-NOCS patients show unacceptable rates of adverse cardiovascular events, that may be controlled, and/or reduced by incretin therapy. Indeed, tailored strategies, including incretin-based therapies, should be considered in the treatment of these patients.

## Abbreviations

FFR: fractional flow reserve
GLP-1: glucagon-like peptide 1
hs-CRP: high sensitivity C-reactive protein
MACE: major adverse cardiac events
M1: macrophages 1 (CD68 cell)
M2: macrophages 2 (soluble-CD163 cell)
Mv-NOCS: multivessel non obstructive coronary stenosis
PCI: percutaneous coronary intervention
PSM: propensity score matching
STEMI: ST elevation myocardial infarction
